# Terahertz Pulsed Imaging and Magnetic Resonance Imaging as Tools to Probe Formulation Stability

**DOI:** 10.3390/pharmaceutics5040591

**Published:** 2013-10-25

**Authors:** Qilei Zhang, Lynn F. Gladden, Paolo Avalle, J. Axel Zeitler, Michael D. Mantle

**Affiliations:** 1 Department of Chemical and Biological Engineering, Zhejiang University, Hangzhou 310027, China; E-Mail: aleizhang@outlook.com; 2 Department of Chemical Engineering & Biotechnology, University of Cambridge, Cambridge CB2 3RA, UK; E-Mails: lfg1@cam.ac.uk (L.F.G.); mdm20@cam.ac.uk (M.D.M.); 3 Merck Sharp & Dohme Ltd., Hoddesdon EN11 9BU, UK; E-Mail: p.avalle@msd.com

**Keywords:** magnetic resonance imaging, terahertz pulsed imaging, formulation stability, drug release, controlled release

## Abstract

Dissolution stability over the entire shelf life duration is of critical importance to ensure the quality of solid dosage forms. Changes in the drug release profile during storage may affect the bioavailability of drug products. This study investigated the stability of a commercial tablet (Lescol^®^ XL) when stored under accelerated conditions (40 °C/75% r.h.). Terahertz pulsed imaging (TPI) was used to investigate the structure of the tablet coating before and after the accelerated aging process. The results indicate that the coating was reduced in thickness and exhibited a higher density after being stored under accelerated conditions for four weeks. *In situ* magnetic resonance imaging (MRI) of the water penetration processes during tablet dissolution in a USP-IV dissolution cell equipped with an in-line UV-vis analyzer was carried out to study local differences in water uptake into the tablet matrix between the stressed and unstressed state. The drug release profiles of the Lescol^®^ XL tablet before and after the accelerated storage stability testing were compared using a “difference” factor *f*_1_ and a “similarity” factor *f*_2_. The results reveal that even though the physical properties of the coating layers changed significantly during the stress testing, the coating protected the tablet matrix and the densification of the coating polymer had no adverse effect on the drug release performance.

## 1. Introduction

Dosage form stability is an important aspect in the drug development processes. Accelerated stability testing allows manufacturers to establish recommended storage conditions and evaluate the shelf-life of products. Changes in drug stability may compromise its efficacy or even risk patient safety [1]. The stability of drug products can be affected by temperature, humidity and light exposure, and products for the global market may need to be evaluated for stability in different climatic conditions [2]. In the drug development process, stability studies are performed in several stages, such as for the stability of the active pharmaceutical ingredients (API) [3], excipients stability [4], formulation stability [5] and packaging stability [6].

For solid dosage forms, direct evidence of product stability is gathered using standard drug dissolution results. Factors such as formulation components, processing and storage conditions can all influence the dissolution stability of tablets during storage. The effects of these factors are largely formulation dependent and may need to be evaluated on an individual basis [7]. Krishnaiah *et al*. [8] studied the stability of a controlled release tablet made of a three-layer guar gum matrix using high-performance liquid chromatography (HPLC) and differential scanning calorimetry (DSC). For this formulation no change in physical appearance, drug content or dissolution profile was found after storage at 40 °C/75% r.h. (relative humidity) for 6 months. In a different study, Nafee *et al*. [9] compared miconazole nitrate dosage forms (sustained release mucoadhensive patches) before and after aging using scanning electron microscopy and a two phase titration technique. They found that the release rate of miconazole nitrate increased after being stored at 37 °C/75% r.h. and this observation was linked to the change of the drug crystallinity: Nafee *et al*. suggested that the drug crystals transformed into an amorphous state during the stressed storage period. In contrast, Rohrs *et al*. [10] have shown that the drug release rate of a delavirdine mesylate instant release tablet decreased upon exposure to high humidity (75% r.h.). In this study a ^13^C CP/MAS NMR technique was used to identify and quantify drug form changes in tablets. The results indicated that the decrease of the drug release rate was related to the structural change of the drug upon contact with moisture. The decrease in drug release rate can be caused by the interaction of moisture and disintegrants, which slow down the disintegration process [11]. The same effect was observed following recrystallization of the drug into a less soluble crystal structure [12]. Tablet coatings and coating materials were found to have a strong influence on the dissolution stability when storing tablets at ICH (International conference on Harmonisation) accelerated stability conditions (at 40 °C and 75% r.h.), which was explained mainly due to moisture barrier effects [13].

With the development and application of non-destructive and non-invasive imaging techniques in the pharmaceutical sciences, spatially resolved information can be used to gain a better understanding of quality, performance and release mechanisms of solid dosage forms [14]. For instance, Ho *et al*. [15] used terahertz pulsed imaging (TPI) to study the coating thickness, homogeneity and quality of tablets produced at lab and pilot scales. They were able to compare the TPI results with the dissolution performance of the tablets, which were coated with a sustained release coating layer. They found a much stronger correlation between the terahertz parameters and the dissolution performance compared to the weight gain and dissolution studies.

Here we use TPI for the first time to characterize the effect of accelerated stress testing (40 °C and 75% r.h.) on the coating of a commercial tablet. To complement the TPI results, 2D magnetic resonance images coupled with in-line UV-vis analysis of cumulative drug concentration were acquired during a subsequent dissolution study to investigate the effect of the aging process on the penetration of dissolution medium into the coated tablet matrix and subsequent drug release.

## 2. Experimental Section

### 2.1. Materials

Samples of commercial Lescol^®^ XL (Novartis, Switzerland) were used for this study. The tablets were coated controlled release systems of cylindrical shape (diameter: 10 mm, thickness: 4 mm), and contained the active component fluvastatin sodium (molecular weight 433.46 g/mol). Each tablet contained 80 mg of fluvastatin sodium. HPMC (K100LV, 30 wt%) was used as the polymer matrix to control the drug release rate. Other main excipients include microcrystalline cellulose (33 wt%), hydroxypropyl cellulose (5 wt%), potassium bicarbonate (2.5 wt%) and povidone (1.5 wt%). The tablet is coated with Opadry yellow which is an film coating composed of hypromellose, titanium dioxide and other ingredients. Deionized water was used as the dissolution medium.

### 2.2. Accelerated Stability Testing Conditions

ICH accelerated tablet stability condition (40 °C and 75% r.h.) was applied in this study. The tablets were kept in a desiccator (100 mm, Fisher Scientific, UK) which contained 100 mL of saturated aqueous solution of sodium chloride (NaCl) in order to maintain an atmosphere of 75% r.h.. The desiccator was sealed with silicone grease and kept in an oven at 40 °C for 4 weeks.

### 2.3. TPI Measurements

**Figure 1:**
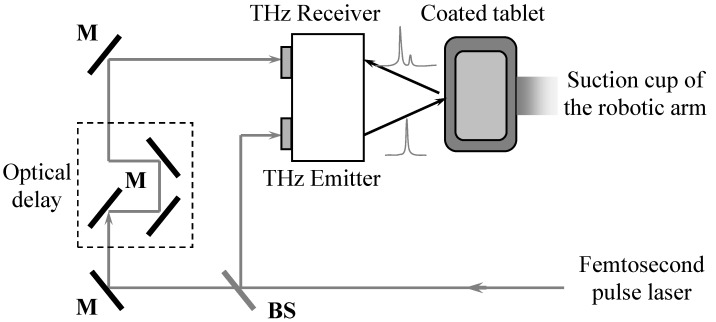
A schematic diagram shows the principle of the TPI imaga2000 system (adapted from [16]). BS: Beam splitter; M: Metallic mirrors.

The TPI studies were performed using a TPI imaga2000 system (TeraView, Cambridge, UK). Images were acquired by point mapping over the entire surface of the tablet. A robotic arm was used to hold the tablet and ensure that for each acquired terahertz time-domain waveform the tablet is kept at angle of normal incidence to the terahertz optics. In order to achieve this, an optical laser gauge is used to scan the topography of the tablet surface and produce a 3D surface map of the entire tablet before each TPI measurement. This information is then used for subsequent TPI mapping. The whole measurement process is fully automated. Figure 1 shows a schematic diagram of the TPI imaga2000 system. Briefly, terahertz radiation is generated by pumping a biased photoconductive antenna with an ultrashort laser pulse from a Ti:Sapphire laser. The emitted terahertz pulse is then focused onto the tablet surface and the reflected and backscattered terahertz pulse is collected and focused onto an unbiased photoconductive antenna for laser-gated coherent terahertz detection [16].

The TPI imaga2000 system has a spatial resolution at surface of 200 µm in lateral direction with a coating layer thickness precision of 2 µm. The layer thickness (axial) resolution is approximately 40 µm. The samples in this study were scanned in a point-to-point mapping mode with a step size of 200 µm in both x and y directions. A single terahertz time-domain waveform can be acquired in approximately 50 ms and it took approximately 60 min to complete a full 3D scan of the entire tablet in this study. The depth of penetration of the terahertz radiation in air was set to 2.5 mm. Coating thickness, peak intensity and interface index were extracted and all of the data were analyzed using TPIview software (TeraView, Cambridge, UK).

### 2.4. Dissolution Processes

The experimental setup used for the MRI studies of the tablet dissolution process follows closely that of [17]. Briefly, a commercial USP-IV dissolution apparatus (Sotax, Switzerland) with an internal diameter of 22.6 mm was integrated into a NMR spectrometer and the dissolution process was studied inside the USP-IV dissolution cell. All experiments were performed on a Bruker AV 400 spectrometer with a vertical 9.4 T superconducting magnet and micro imaging facilities. A 25 mm dual resonance birdcage radio frequency (r.f.) coil operating at 400.22 MHz for proton (^1^H) was used. The “T2-preconditioned Rapid Acquisition with Relaxation Enhancement (RARE)” pulse sequence [18] was used in this study to acquire quantitative water concentration maps. The images were acquired on the radial plane with a field-of-view of 24 mm × 24 mm and a slice thickness of 1 mm. A 64 × 64 data matrix gives an in-plane resolution of 0.375 mm × 0.375 mm. A phase encoding start value of −0.22 was chosen to maximize the quantitative nature of the images in accordance with the work of Chen *et al*. [18]. All images were acquired in the split echo acquisition mode and the acquisition of a single group of images at even echo times of 18.87, 20.87, 26.87, 50.87, 82.87, 146.87, 274.87, 530.87 ms and odd echo times of 18.55, 20.55, 26.55, 50.55, 82.55, 146.55 ms, 274.55, 530.55 ms. The repetition time was 10 s and signals were averaged over 2 repetitions. Three replicate dissolution runs were acquired for each the stressed and the unstressed condition.

### 2.5. Dissolution Protocol

The dissolution was performed in a USP-IV flow-through cell operated in a closed configuration. Deionized water was used as the dissolution medium and the temperature in the USP-IV cell was conditioned to 37°. Liquid flow at a volumetric liquid flow rate of 8 mL/min was achieved using a peristaltic pump (205S, Watson Marlow, Falmouth, UK).

### 2.6. UV Measurements

The drug release rate was measured on-line using a UV-Vis spectrophotometer (HP 8452A, Hewlett Packard, Bracknell, UK). Samples of 4 mL were withdrawn from the bulk solution at predetermined times and analyzed at a wavelength of 303 nm. After each measurement, the sample was poured back into the bulk solution.

## 3. Results and Discussion

### 3.1. Results

#### 3.1.1. Coating Characterization

**Figure 2:**
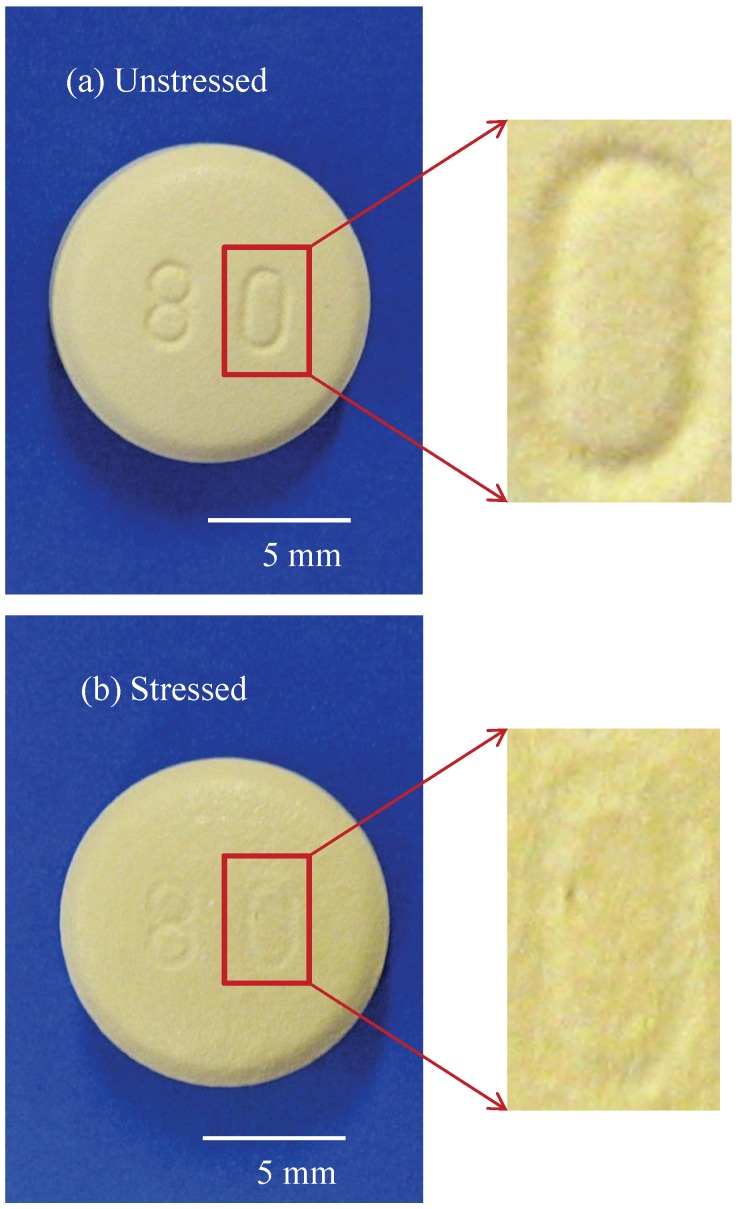
Comparison of the surface morphology of two Lescol^®^ XL tablets stored in (**a**) unstressed and (**b**) stressed conditions. Stressed condition: 40 °C/75% r.h., 4 weeks; Unstressed condition: sealed plastic container (the original packaging) at room temperature

Figure 2 shows an optical image highlighting the surface morphology of two tablets before and after storage in the stressed conditions. The stressed tablet was stored in accelerated conditions (40 °C/75% r.h.) for four weeks and the unstressed tablet was kept in its original sealed plastic bottle at room temperature (*ca.* 20 °C). The digital pictures shown in Figure 2 highlight the fact that there was an observable difference between the surfaces of the two tablets. The unstressed tablet exhibited a slightly lighter color with sharp and clear embossed letters, whereas the stressed tablet had a rougher surface and blurred embossed letters. This is reflected in the coating thickness variation as measured by TPI.

**Figure 3:**
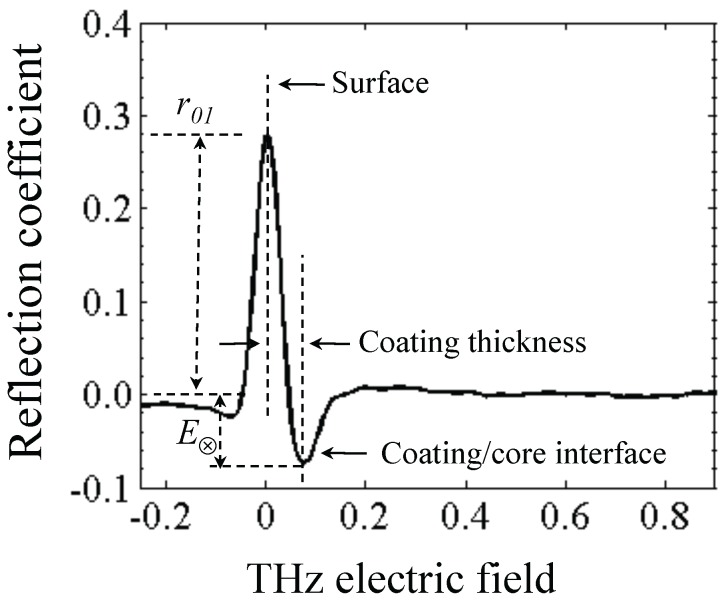
A typical time-domain terahertz waveform acquired via TPI measurements from an unstressed Lescol^®^ XL tablet.

Figure 3 shows a typical time-domain terahertz waveform acquired via TPI measurements from an unstressed Lescol^®^ XL tablet. The magnitude of the maximum reflection peak shown in Figure 3 at a penetration depth of 0 mm corresponds to the reflection coefficient (*r*_01_) as part of the THz pulse reflects from the interface between air and the coating film, whereas the peak that shows a negative reflection coefficient (*r*_12_) indicates the reflection from the interface between the coating film and the tablet core. The negative value of *r*_12_ indicates that the refractive index of the coating film (*n*_1_) is higher than that of the tablet core (*n*_2_).

The coating thickness of the tablet was derived by analysis of the time-domain terahertz waveform at each sampling point.

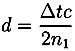
(1)
where *d* is the coating thickness, Δ*t* is the time delay between the two interface reflection peaks, *c* is the speed of light and *n*_1_ is the refractive index of the coating film.

**Figure 4:**
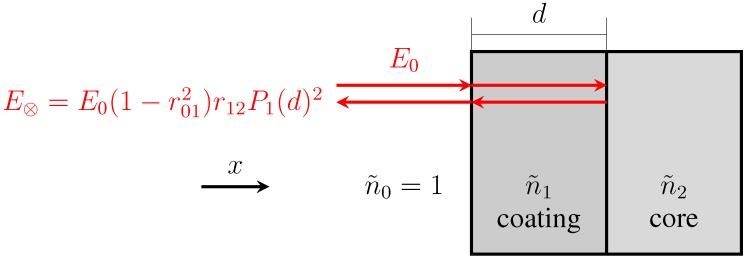
Schematic of a basic model of the propagation of electromagnetic radiation into a single coating layer of thickness *d* with refractive index *ñ*_1_ from free space (refractive index *ñ*_0_). The tablet core is characterized by a refractive index of *ñ*_2_. The terahertz pulse propagates from the source (not shown, to the left of the sample) to the detector (not shown, also to the left of the sample) along *x*.

The reflection coefficients (*r*_01_, *r*_12_) from the two interfaces are measured via the time-domain terahertz waveform, and the data can be used to calculate the refractive indices of the coating film and the tablet core, as shown in Equations (2) and (3). 

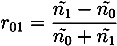
(2)

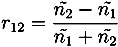
(3)
where *ñ*_0_, *ñ*_1_ and *ñ*_2_ are the refractive index of air, coating film and tablet core, respectively. Note that the simple implementation of Fresnel’s equation in Equation (3) does not account for any reflection losses at the surface [16]. In order to achieve this we need to consider the propagation of the terahertz pulse as outlined in Figure 4.

Here *P* is the material interaction term: 

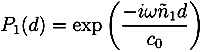

with *ñ*_1_ = *n*_1_ + *iκ*_1_
and 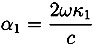
(4)
where *κ* is the extinction coefficient and *n* the real refractive index of the material. Given the low absorption of typical polymers used in film coating (on the order of *κ* ≈ 0.006) as well as the low film thickness we can neglect the term *P*_1_ and derive the following expression for the amplitude of the reflection between coating and core *E*_⊗_ based on the real refractive indices

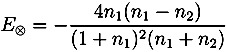
(5)
which we can solve for *n*_2_

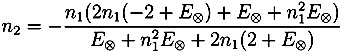
(6)


By mapping over the entire surface of the tablets (top, bottom and central surfaces) 3D maps of the tablet can be acquired. From this dataset the spatial distribution of the coating thickness, peak intensity and interface index can be extracted. The statistical distributions of these parameters can also be quantified. Here, peak intensity (also referred to as terahertz electric field peak strength, TEFPS) is defined as the magnitude of the reflection coefficient from the tablet surface relative to the reference reflection from a mirror. The interface index is defined as the magnitude of the reflection coefficient from the interface between the coating and the inner tablet normalized to the reflection coefficient from the interface between the coating and the surface. Both parameters can be derived from the time-domain terahertz waveform according the Equations (7) and (8) [19]: 

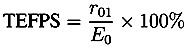
(7)

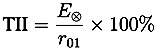
(8)
where TEFPS is the peak intensity, *E*_0_ is the amplitude of the reference incident terahertz pulse, TII is the terahertz interface index. Both parameters are strongly affected by the refractive index of tablet coating layer and can be used to provide information on relative coating density over the surface of the tablet. The interface index may also denote the changes in the physicochemical properties at the interface between the coating and the inner tablet.

**Figure 5:**
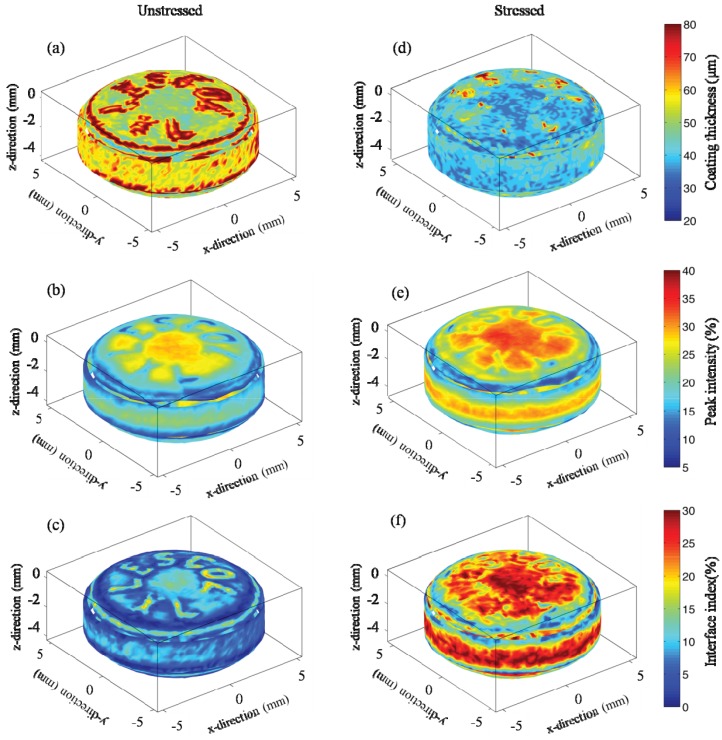
Typical 3D maps of coating thickness, peak intensity and interface index of Lescol^®^ XL tablets acquired by TPI measurements. (**a**)–(**c**): Coating thickness, peak intensity and interface index of an unstressed tablet; (**d**)–(**f**): Coating thickness, peak intensity and interface index of a stressed tablet.

Figure 5 shows typical 3D maps of coating thickness, peak intensity and interface index of an unstressed and a stressed Lescol^®^ XL tablet acquired by TPI. Table 1 lists the refractive indices and coating thickness values calculated from the top, bottom and central surfaces of these two tablets using Equations (1)–(3). It is important to note that the embossed areas of the tablet surfaces have distinctive but unreliable coating thickness results due to strong scattering as a result of the strong curvature in those regions [20]. These data points were filtered out by thresholding and not used in the subsequent data analysis. The two coating thickness maps in Figure 5 show that the unstressed tablet had clear and distinctive embossed letters on the top surface, whereas the embossed letters on the stressed tablet surface became blurred and unrecognizable. This observation matches with the visual appearance of the tablets (Figure 2). Moreover, Table 1 shows that the coating thickness of the unstressed tablet is approx. 10 µm thicker than that of the stressed tablet, and the refractive index of the coating film (*n*_1_) increased from approx. 1.5 to 1.7, whereas the refractive index of the tablet core immediately below the coating layer (*n*_2_) increased from approx. 1.6 to 1.9. Figure 5 shows that the average peak intensity and interface index of the stressed tablet was higher than that of the unstressed tablet. The results indicate that the coating polymer undergoes a restructuring process and that the tablet matrix below the coating is also affected by the stress conditions. From this analysis alone, it is unclear how deep this change in the tablet extends into the matrix and whether it has any impact on the drug release characteristics.

**Table 1:** Refractive indices and coating thickness of the tablets in Figure 5. *r*_01_ and *E*_⊗_ are the amplitudes of the reflection peaks [as defined in Figure 3 and Equation (6)] of the air/coating and coating/core interfaces respectively; *n*_0_, *n*_1_, *n*_2_ are the refractive index of air, coating film and tablet core, respectively; *d* is the coating thickness of the tablets, that was calculated using the time delay and the respective refractive index, *n*_1_.

		*r*_01_	*E*_⊗_	*n*_0_	*n*_1_	*n*_2_	*d*/µm
Unstressed	Top	0.21	-0.01	1.00	1.54	1.57	50.6
	Bottom	0.22	-0.01	1.00	1.55	1.58	49.9
	Centre	0.18	-0.01	1.00	1.43	1.46	57.3
Stressed	Top	0.26	-0.05	1.00	1.70	1.89	41.0
	Bottom	0.26	-0.06	1.00	1.72	1.89	43.1
	Centre	0.25	-0,06	1.00	1.67	1.90	40.2

**Figure 6:**
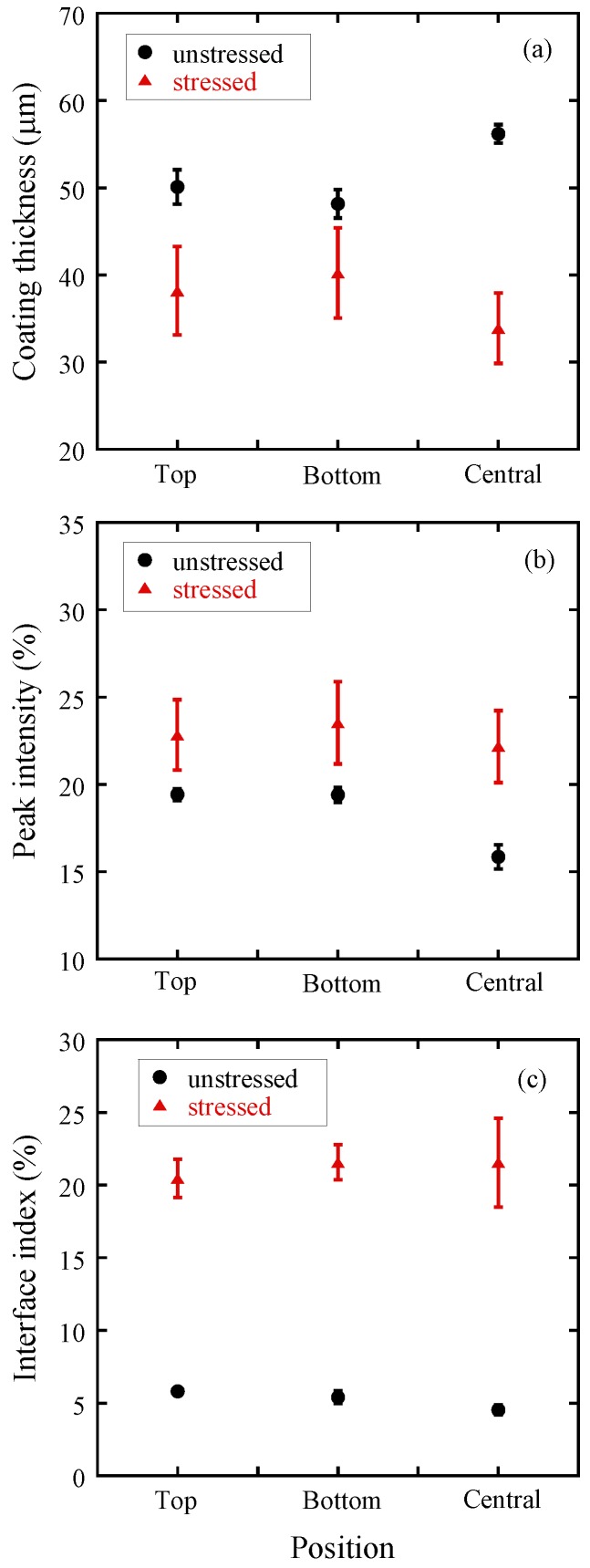
Statistical values of terahertz parameters at different surface positions of Lescol^®^ XL tablets. (filled circles) Unstressed tablets; (filled triangles) Stressed tablets. (**a**) Average coating thicknesses; (**b**) Average peak intensity; (**c**) Average interface index. The data were Error bars are the standard deviation.

Statistical data of coating thickness, peak intensity and interface index of stressed and unstressed tablets averaged from six tablets each are presented in Figure 6. The results show that for the unstressed tablet, the variation of the coating thickness on the three surfaces was minimal and the average coating thickness of the whole tablet was approximately 50 µm. After the tablet was stressed under accelerated condition for 4 weeks, the coating thickness decreased on all three surfaces resulting in an average coating thickness of ≈40 µm, which is close to the minimum measurable thickness of this technique (in the range of 30–40 µm) [21,22]. Figure 6 shows that the average peak intensity of the unstressed tablet is below 20%. However, the average peak intensity of the stressed tablets is ≈23%. Moreover, the results show a dramatic change of the interface index after stressing. The average interface index values changed from 5% (unstressed tablets) to 20% (stressed tablets). Figure 6 shows that the standard deviation of the statistical values from the stressed tablets is significantly higher than that from the unstressed tablets.

**Figure 7:**
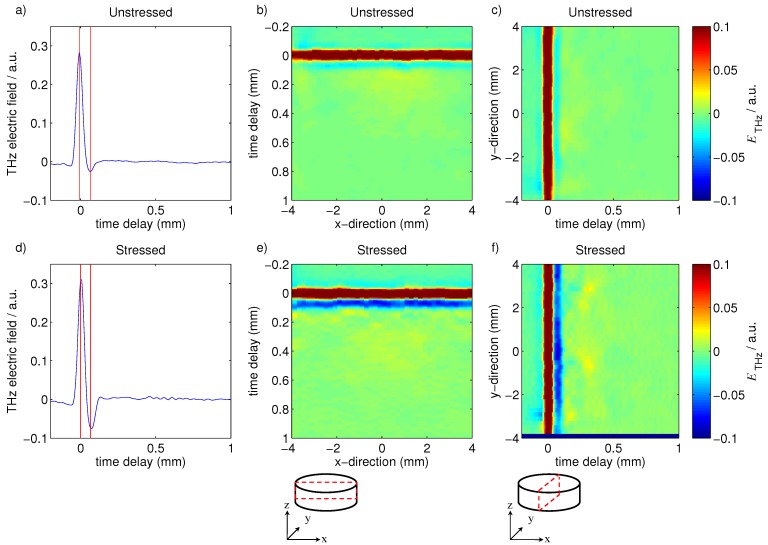
Typical terahertz cross sections acquired by TPI measurements. Unstressed tablet: (**a**) Waveform, the coating layer is defined by the time delay between the first maximum and the subsequent minimum using Equation (1) as marked by the red lines; (**b**) Virtual cross section (B-scan) in *x*-direction. In this representation the air/coating interface is represented in dark red and the coating/tablet interface is represented in light/dark blue; (**c**) B-scan in *y*-direction. Stressed tablet: (**d**) Waveform; (**e**) B-scan in *x*-direction; and (**f**) B-scan in *y*-direction.

As the point-to-point imaging mode of the TPI measurement acquires a terahertz waveform of each pixel, and the terahertz pulse can penetrate ≈2 mm deep into tablets [14], virtual cross sections in the *x*, *y* and *z* directions can be extracted with this technique to detect changes of the tablet internal structure during the storage process. Figure 7 shows typical virtual cross sections of the unstressed and stressed Lescol^®^ XL tablets. The dark red bands shown on these images are the interface between air and the coating films, and the light and dark blue bands that immediately follow the dark red bands are the coating/tablet core interface. The light green/yellow areas are the tablet core regions. The results show that the stressed tablet had much higher reflectivity in the coating/core interface, which is due to the increase of the refractive index of the coating film after stress testing. Figure 7 also shows that the unstressed tablet had a relatively uniform profile in the tablet core region, whereas the stressed tablet presented a slight increase of reflection coefficients in the tablet core area, especially the region close to the coating/core interface. It seems that the accelerated storage test can affect the tablet core matrix up to a depth of <0.4 mm. For a depth of >0.4 mm, the stressed tablet showed a similar profile compared to the unstressed tablet.

#### 3.1.2. Tablet Dissolution Testing

**Figure 8:**
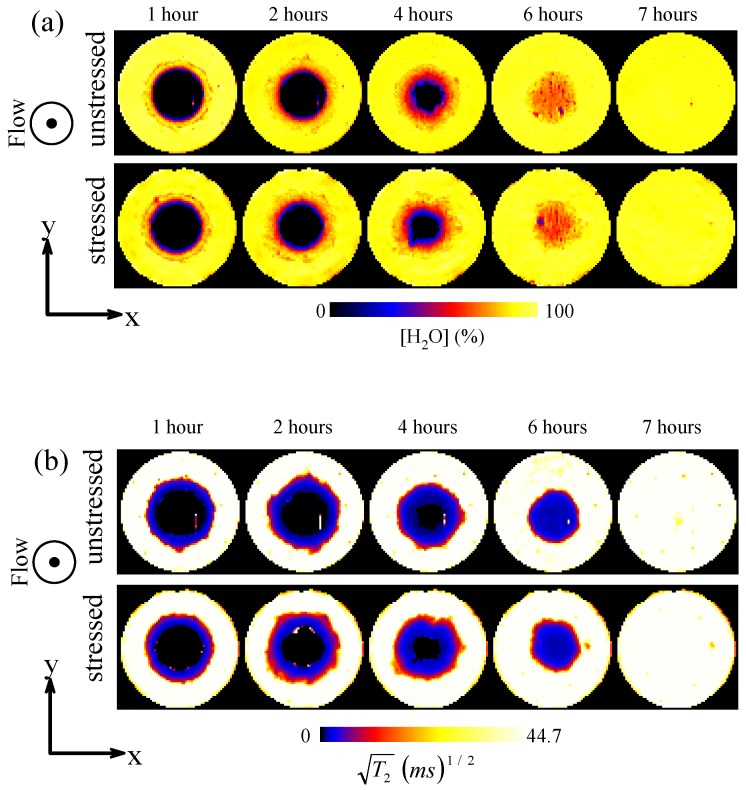
Typical ^1^H MRI images showing the tablet dissolution processes in water. (**a**) Water concentration maps of the unstressed and stressed Lescol^®^ XL tablets; (**b**) *T*_2_ relaxation maps of the unstressed and stressed Lescol^®^ XL tablets.

The dissolution processes of the unstressed and stressed tablets were compared using both ^1^H MRI images and the in-line drug release profiles. Figure 8 shows some typical ^1^H MRI images illustrating the gel formation process of the tablets. Here images were acquired from the *xy*-plane and the water concentration maps (Figure 8a) show the water concentration in the gel layer at different time points, whereas the *T*_2_ relaxation maps (Figure 8b) show the gel structure of the tablets during the dissolution processes. Figure 8 shows that the unstressed and stressed Lescol^®^ XL tablets have comparable gel formation processes. The erosion of the gel surface became apparent in the images after *t* = 2 h. The tablets were totally penetrated by water between 4 to 6 h and both of the tablets were fully dissolved at approximately 7 h.

**Figure 9:**
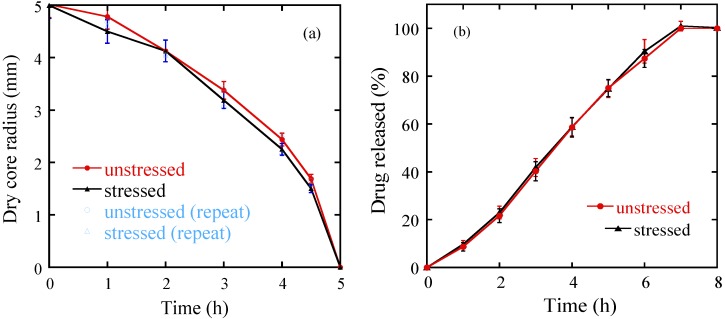
(**a**) Dry core shrinkage profiles of the unstressed and stressed tablets during dissolution. Data from an experimental repeat: (open circles) unstressed tablets; (open triangles) stressed tablets. Radius data are averaged from 4 radial directions and the error bars are the standard deviation of data from these 4 repeats. (**b**) Drug release profiles of the unstressed and stressed tablets in the dissolution processes. MRI and dissolution were studied using three tablet samples each.

Figure 9a shows the tablet dry core shrinkage profiles which reflect the water penetration rates in the unstressed and stressed tablets. The dry core is defined based on the images in Figure 8b. First, all of the images were pre-gated with a threshold value equal to five times the value of the standard deviation of the background noise to remove noise in the image. The area within the tablet with value equal to 0 is defined as the dry core. The results show that the unstressed and stressed Lescol^®^ XL tablets (Figure 9a) have similar dry core shrinkage profiles during the dissolution processes. The on-line drug release profiles of these tablets are shown in Figure 9b. The results show that the unstressed and stressed Lescol^®^ XL tablets have almost identical drug release curves, which indicates that the accelerated storage condition does not affect the drug release process of the Lescol^®^ XL tablet.

### 3.2. Discussion

#### 3.2.1. The Effect of Storage on Tablet Coating in Lescol^®^ XL

Coating is commonly applied to oral dosage forms in order to achieve specific functions such as controlling drug release processes and enhancing dosage form stability [23]. Humidity and/or elevated temperature may drastically alter the coating properties when the dosage form is subjected to accelerated storage conditions, which may have an effect on drug release processes [7]. The results shown in this study indicate that the direct exposure of the Lescol^®^ XL tablet to the accelerated storage condition changed some coating properties, such as coating roughness and thickness. Stressed Lescol^®^ XL tablets show rougher appearance after being stored at 40 °C/75% r.h. for 4 weeks (see Figure 2).The physical appearance of the stressed tablet shown in Figure 2 and also the TPI images shown in Figure 5 reveal that the embossed letters on the tablet surface became blurred and less distinct after storage. This can be seen as evidence for the existence of a rearrangement process of the coating materials during the storage process.

The 3D TPI images in Figure 5 show that the coating thickness decreased after storage. One of the possible reasons that cause the decrease of the tablet coating thickness is the relaxation/rearrangement of the coating materials. Table 1 listed that the refractive index of the coating film (*n*_1_) increased from approx. 1.5 to 1.7, which is an indication of variation in density of the coating film. The decrease of the coating thickness and the increase of the refractive index of the coating layer indicate that the accelerated storage test resulted in a densification of the coating layer. This is likely due to a rearrangement of the polymer as a result of higher mobility of the polymer material during the accelerated stability testing conditions, similar to a densification process during the curing process. Murthy *et al*. [7] discussed the re-crystallization and hardening processes of sugar coated controlled release tablets under accelerated storage conditions. The sugar coating first dissolved when exposed to a combination of high humidity and temperature and subsequently recrystallized and hardened. Here, the film coating contains polymers like hypromellose, and these polymer chains may relax/mobilize when the tablet is exposed to a combination of high temperature and high humidity. This could initially cause the coating to increase thickness due to the formation of a gel-like layer. However, in this study the tablets were transferred into sealed plastic bags and stored at room temperature for one week after the storage period. It is possible that the coating materials subsequently re-crystallized or hardened in the post-storage period (curing), which reduces the coating thickness of the tablets. Gendre *et al*. [24] used X-ray micro-computed tomography to study the curing process of a tablet coating layer. The results revealed a densification of the coating layer with a decrease of the overall coating thickness by about 10 µm. The terahertz results obtained here are consistent with the results reported by these authors. This hardening process of the tablet coating can be proven by the changes of surface intensity and interface index obtained from the TPI measurement. In terahertz measurements, the peak intensity and interface index of a tablet coating has a strong correlation with the density or hardness of these coating films [25,26]. Research has shown that higher peak intensity and interface index values are possible indications of denser or harder tablet coatings when comparing similar dosage forms [15,19].

In addition to the changes of the coating layer, Figure 7 shows that the inner tablet core areas that are close to the coating layer (depth <0.4 mm) is likely to have certain variation after storage under accelerated stress conditions. However, the effect on the tablet core is limited as the majority of the inner core areas with depth >0.4 mm showed similar TPI profiles compared to that of the unstressed tablet. This is also reflected in the change in *n*_2_, the refractive index of the tablet matrix immediately below the polymer coating layer (Table 1). We observed a marked increase in *n*_2_ upon storage under stress conditions. This further demonstrates that moisture can penetrate into the tablet core matrix, albeit in very limited quantity given that the change in the microstructure cannot be detected for a depth >0.4 mm (Figure 7). The moisture clearly results in restructuring processes within the tablet matrix below the coating. This process results in localized clusters of higher polymer density (red spots in Figure 7c,d).

To summarize, the coating of Lescol^®^ XL tablets became thinner and harder. However, it is important to asses whether the change of the tablet coating layer and subsequent polymer matrix does affect the tablet dissolution and drug release processes.

#### 3.2.2. The Effect of Storage on Dissolution Processes

The dissolution data obtained from both the unstressed and stressed tablets is useful in evaluating the ruggedness of the tablets and its ability to withstand the variation of conditions during transport and storage [7]. The results shown in this study indicate that the Lescol^®^ XL tablet has good dissolution stability over a four-week storage period under accelerated conditions (see Figure 8 and Figure 9). In order to quantify the differences between the drug release, a mathematical calculation [26] using factors known as *f*_1_ and *f*_2_ can be applied:

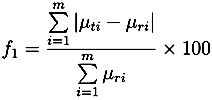
(9)

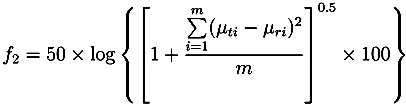
(10)
where *f*_1_ is referred to as the “difference” factor and *f*_2_ is the “similarity” factor, *m* is the number of time points, *µ_ri_* is drug release value of the unstressed tablet, *µ_ti_* is drug release value of the stressed tablet and log is the decadic logarithm.

The two equations were proposed by Moore and Flanner [27] and implemented by the US Food and Drug Administration (FDA). Generally, values between 0 to 15 for *f*_1_ and 50 to 100 for *f*_2_ indicate similarity or equivalence of the two drug release curves [28].

Table 2 shows the results of *f*_1_ and *f*_2_ for the Lescol^®^ XL tablets. The “difference” factor *f*_1_ is close to 0 and the “similarity” factor *f*_2_ is almost 100, which indicates that the difference between the release curves of the stressed and unstressed tablets are negligible.

**Table 2:** Difference (*f*_1_) and similarity (*f*_2_) factors of the stressed and unstressed tablets.

	Lescol^®^ XL
*f*_1_	2.1
*f*_2_	99.9

#### 3.2.3. The Effect of the Coating Layer on Drug Release

The reason why the Lescol^®^ XL tablets have consistent dissolution behavior before and after storage can be explained by the fact that the coating layer limits the amount of moisture that can penetrate into the tablet matrix [13]. Castellanos Gil *et al*. [29] compared the stability of HPMC tablets uncoated and coated with copovidone under 25 °C/75% r.h. for two years. The results showed that the coating on the surface of the tablets reduced the amount of absorbed water from the atmosphere and increased the dissolution stability when compared to the uncoated tablets. The coating on the Lescol^®^ XL tablets is likely to be an important factor to enhance the tablet stability when exposed to elevated temperature and humidity conditions (40 °C/75% r.h.). Our results show that although the coating thickness and density changes over the storage period the majority of the internal core remains unchanged based on the TPI data and the moisture penetration behavior as analyzed by MRI. However, Figure 7 shows that the tablet matrix that is close to the coating layer exhibited a slight increase of reflection coefficients compared to the unstressed tablet, which may indicate that a limited amount of moisture has interacted with the tablet matrix just below the coating layer. It is possible that even higher temperature or humidity, or longer storage time may change the structure of the internal core further. However, the MRI and dissolution data clearly showed that under the conditions chosen in this study the change of the coating layer did not have any effect on the drug release kinetics and it seems that the coating layer successfully fulfilled its role to stabilize the dosage form over the shelf life.

## 4. Conclusions

The dissolution stability of the Lescol^®^ XL tablet was studied under accelerated storage conditions (40 °C/75% r.h.). TPI was applied to characterize the coating properties of the Lescol^®^ XL tablet before and after stress testing and the results indicate that the technique is a useful analytical tool in spatially mapping the coating thickness and density. The TPI results show that the thickness of the tablet coating decreased after storage at elevated temperature and humidity while the density of the tablet coating increased. The dissolution results indicate that the Lescol^®^ XL matrix is sufficiently protected by the coating even though the coating does not act as an absolute barrier for moisture. The combination of TPI and MRI provides a useful research method for examining the effects of stress testing of pharmaceutical formulations.
